# Experiments on the Blood

**Published:** 1845-11

**Authors:** 


*Experiments on the Blood.* By M. Dupuy.—The following experiments show how very rapidly the blood alters in its qualities, and M. Dupuy thinks they tend to throw light on the development of malignant carbuncle, plague, &c.

A few ounces of arterial blood were drawn from the carotid artery of a horse, and found to contain twenty-one grains of moist fibrin per ounce. The pneumogastric nerves were then included in a ligature, and in a few minutes afterwards a few more ounces of blood were drawn from the same artery. It was found to have assumed a black colour, like venous blood, and to contain much less fibrin.

The same experiment was performed on another horse, with this difference, that the ligature was only so far tightened as to compress the nerve without destroying its texture. The arterial blood was black like venous blood, a few seconds after the nerve was compressed; but almost immediately assumed its bright arterial hue when the ligature was relaxed. This curious experiment was frequently repeated on the same animal in the presence of MM. Thouret, Halle, and Dupuytren.

Another horse, one ounce of whose arterial blood yielded twenty-one grains of moist fibrin, had tracheotomy performed, and then the pneumogastric nerves cut across. On the sixth day thereafter, an ounce of its arterial blood yielded only seven grains of moist fibrin. It died on that day. The cut ends of the nerves were found on the third day to be inflamed and swollen, and to exhale a very fetid odour. On the fourth and fifth days the respiration was difficult, and the animal presented the same morbid phenomena as those which attend carbuncle or malignant pustule. A portion of the spleen of this animal was placed as a seton under the skin of a vigorous horse. Painful œdematous swelling soon manifested itself, and rapidly extended ; great difficulty of respiration came on, and the animal died on the fifth day with symptoms of asphyxia. The nerves of the eighth pair were found ecchymosed and altered in structure, and the whole symptoms presented a striking analogy with those described by Chaussier as characterising the malignant pustule.

During a series of experiments on injecting various fluids into the veins, it was found that a solution of the healthy cerebral matter of a cow or sheep, suspended in water and injected into the veins of a horse, killed it as instantaneously as a solution of corrosive sublimate, and the morbid appearances were the same.—*Ibid., from Bulletin de l'Academie Royale de Medicine.*

